# Diabetic encephalopathy: metabolic reprogramming as a potential driver of accelerated brain aging and cognitive decline

**DOI:** 10.3389/fcell.2025.1701406

**Published:** 2025-11-17

**Authors:** Jia-xuan Huai, E-e Chang, Yi-ran Zhu, Wen-ling Ma, Tian-su Lv, Jing Sun, Xi-qiao Zhou

**Affiliations:** 1 Department of Endocrinology, Jiangsu Province Hospital of Chinese Medicine, Affiliated Hospital of Nanjing University of Chinese Medicine, Nanjing, China; 2 The First School of Clinical Medicine, Nanjing University of Chinese Medicine, Nanjing, China

**Keywords:** diabetic encephalopathy, metabolic reprogramming, brain insulin resistance, oxidative stress, mitophagy, glycolytic flux, GLP-1 receptor agonists, NAD⁺ boosters

## Abstract

Diabetic encephalopathy (DE) is a serious neurological complication of diabetes and is expressed as progressive decline in cognitive function, emotional disorders, and changes in brain structure. This review brings together the relevant evidence and demonstrates that metabolic reprogramming, the adaptive reconfiguration of the core metabolic pathway in response to hyperglycemia, is a potential driver of accelerated brain aging in DE. The main pathological characteristics are: abnormal brain insulin signaling, resulting in a decrease in neuronal glucose intake and a decrease in mitochondrial oxidative phosphorylation, oxidative stress and neuroinflammation caused by high blood sugar, in which excess reactive oxygen species (ROS), impairs mitochondrial integrity and leads to activation of microglia cells. The impaired mitophagy and the macrophages remove defects and cause the accumulation and energy collapse of the dysfunctional organelles. In addition, it promotes excessive glycolytic flux, lipolysis disorder, lactic acid accumulation, and ceramide-dependent synaptic damage. We further examine shared metabolic mechanisms between DE and neurodegenerative diseases such as alzheimer’s disease (AD) and treatment strategies for pathological metabolic reprogramming including GLP-1 receptor agonists, NAD^+^ boosters, and AMPK activators. This analysis laid the foundation for new intervention measures against the development of DE.

## Introduction

Diabetes mellitus (DM) is one of the most common chronic metabolic diseases in the world and poses a serious threat to human health (American Diabetes Association ProfessionalPractice Committee, 2024; GBD, 2021 Diabetes Collaborators, 2023). Within the scope of diabetic complications, DE is a particularly heavy neurological expression, and its adverse effects on patient health and the lack of current evidence therapy have led to increased interest in research (Chen et al., 2018). Clinically, DE is characterized by progressive cognitive impairment, as a result of memory and performance impairment, emotional disorders including anxiety and depression, and abnormal brain imaging results such as white quality high signal, decreased brain capacity and hippocampal atrophy (Ehtewish et al.), (Mogi and Horiuchi, 2011; Duarte, 2015). DE patients show distinctive brain changes including vascular lesions, demyelinating cranial nerve, and neuronal degeneration, leading to impaired cognitive performance (Mijnhout et al., 2006; Wang et al., 2020). However, the pathogenesis of DE is not fully understood. Previous studies have shown that it is associated with a variety of pathologic processes, including hyperglycaemia, alterations in brain insulin signaling, cerebrovascular disease, excessive phosphorylation of tau protein, neuroinflammation, and oxidative stress (Nagayach et al., 2024; Rösen et al., 2001). Thus, metabolic reprogramming is considered to be a potential core-driven mechanism that connects these elements. However, the molecular cascade of driven DE is only a partial description, and the generally accepted diagnostic criteria have not yet been established.

Metabolic reprogramming represents the basic cellular process in which neurons dynamically adjust the core metabolic pathways, including glucose utilization, mitochondrial respiration, and biomolecular synthesis, to meet the wavy energy demands and challenges of the microenvironment (Wu et al., 2023). Under physiological conditions, astrocytes maintain neuronal metabolic demand by aerobic glycolysis, and lactic acid is an important energy substrate for synaptic function and cognitive expression (Hui et al., 2017; Xiong et al., 2025). However, chronic hyperglycemia leads to inappropriate metabolic recombination. In a mouse model of streptoazobin-induced hyperglycemia, elevated glucose levels translate the phenotype of astrocytes into a proliferative pro-inflammatory state (Zhang et al., 2024). In a model of intellectual disability, a parallel mechanism in which SNX 27 mutations impaired glial glucose intake through glucose transporter 1 (GLUT1), reduced lactic acid production, and converted steady-state glial cells to a reactive state appeared (Lee et al., 2024). Therefore, mechanisms designed for neuroprotection instead accelerate neurodegeneration through self-reinforcing metabolic failure (Bizon et al., 2012).

This review brings together existing evidence and systematically describes the role of metabolic reprogramming in accelerating DE brain aging, including brain insulin signaling dysregulation, oxidative stress and neuroinflammation, autophagy and mitophagy dysfunction, glycolytic Flux and lipolysis Dysregulation. It focuses on abnormal pathways such as disintegration. We also explored common metabolic mechanisms between neurodegenerative diseases such as DE and AD, and developed therapeutic strategies for metabolic reprogramming such as GLP-1 receptor agonists, NAD^+^ boosters, and AMPK activators. Through this analysis, we provided a new theoretical basis and treatment plan for the prevention and treatment of DE.

## Brain insulin signaling dysregulation

The brain is an important target for insulin action, and most brain insulin comes from circulating pancreatic insulin rather than local synthesis. Insulin integrates the functions of metabolism, neuronutrition, neuroregulation and neuroendocrine regulation in the brain and regulates processes closely related to cognitive health. These processes include synaptic plasticity, neurogenesis, and memory consolidation, especially in the hippocampus (Rask-Madsen and Kahn, 2012; Kleinridders et al., 2015). These functions are mediated by the insulin receptors (IRs), which is widely distributed in presynaptic and postsynaptic neurons and neurologlia cells, allowing insulin to modulate neurotransmitter release, receptor transport, and neuronal excitability (Cai et al., 2018; Scapin et al., 2018).

Insulin signaling in the brain has initiated a series of events that are important for neuronal survival and metabolic balance. The binding of insulin to IR activates the receptor tyrosine kinase, leading to phosphorylation of IRs proteins. This fires two pathways: the phosphatidylinositol 3-kinase (PI3K)/Akt cascade and the mitogen-activated protein kinase (MAPK) pathway (Lee et al., 2011). The PI3K/Akt pathway controls glucose intake via glucose carriers, protein making, and cell survival. Glucose enters the brain barrier via GLUT1. Neurons take glucose via GLUT3; astrocytes via GLUT1 (Peng et al., 2021). The MAPK pathway manages nerve connections and new brain cells, key for learning and memory. Insulin also changes CREB protein action, a factor vital for memory fixing (Castrén and Monteggia, 2021). Together, these paths keep brain metabolism and function steady. Their failure in T2DM breaks this balance, pushing bad metabolic reprogramming.

A sign of T2DM is insulin resistance. Cells respond poorly to insulin’s metabolic actions. This reaches the brain as neuron insulin resistance (Muscogiur et al., 2022). In cells, neuronal insulin resistance impairs glucose uptake mechanisms, cell survival, and disrupts the steady state of metabolism. Critically, insulin’s cognitive effects may operate through non-metabolic pathways such as synaptic receptor trafficking and tau phosphorylation regulation, independent of direct glucose uptake (Hamer et al., 2019). Neuronal insulin resistance triggers metabolic reprogramming, shifting energy metabolism from active phosphorylates to glycolysis. This transfer is accompanied by mitochondrial dysfunction and lipid and amino acid metabolism. These metabolic changes accelerate neuronal and brain aging (Cunnane et al., 2020). Biomarkers of brain insulin dysfunction in DE patients reflect the severity of metabolic reprogramming and cognitive impairment. These include reduced IRS-1 phosphorylation, changes in mitochondrial morphology/function, and malfunctioning of downstream insulin signaling targets (Martínez Báez et al., 2024). These biomarkers not only tested the link between insulin resistance and DE, but also emphasized that metabolic reprogramming is a unified mechanism by which T2DM accelerates brain aging.

## Oxidative stress and neuroinflammation

Oxidative stress is defined as the imbalance between the production of ROS and the antioxidant defense capacity of an organism, and is a central pathological feature of metabolic disorders, neurodegeneration and aging (Maciejczyk et al., 2019). Importantly, it is the result of metabolic reprogramming, which acts as an adaptive restructure of metabolic pathways. Sustaining accelerated brain aging and cognitive decline of DE. Antioxidative systems of the body containing catalase, superoxide dismutase, enzymes such as glutathione peroxidase, and non-enzymatic components such as reduced glutathione and uric acid, usually works to neutralize ROS and maintain cell homeostasis (Chen et al., 2025). However, in the condition of chronic diabetes, oxidative stress can overwhelm these defense mechanisms, lead to impaired regulation of metabolic pathways, and cause pathological changes in energy metabolism that perpetuate cellular dysfunction (Singh et al., 2022).

DM contributes greatly to excessive ROS production. Chronic hyperglycemia in DM accelerates ROS formation, which overcomes endogenous antioxidant defenses, through glucose self-oxidation, advanced glycemic reactions, and extreme activity of the polyol pathway (Khalid et al., 2022). In diabetic rats, weak brain antioxidant enzymes match worse memory problems (Ling et al., 2018). Specifically, ROS harm to mitochondrial DNA and parts blocks energy making (Sun et al., 2023; Watanuki et al., 2024). This forces neurons to use inefficient sugar breakdown for energy—a mark of metabolic reprogramming. This sugar shift cuts ATP output and raises lactate buildup and more ROS. It creates a bad loop that speeds neuron aging (Vargas-Soria et al., 2023; Churchward et al., 2018).

High ROS levels also push microglia cells to change. They release stuff like TNF-α, IL-1β, IL-6, IFN-γ, iNOS, MCP-1, NO, prostaglandin E2, and GRO, increasing brain swelling (Bernier et al., 2020). Brain samples from diabetic donors show microglia activation, like in AD—a zone key for memory (Valente et al., 2010). Pre-clinical studies using diabetic/obese db/db mice have shown that increases in brain levels of IL-1β, IL-6, and TNF-α are correlated with decreases in spatial recognition memory. However, genetic or pharmacological inhibition of microglial activation can mitigate both inflammation and cognitive impairment (Din et al., 2011).

**FIGURE 1:**
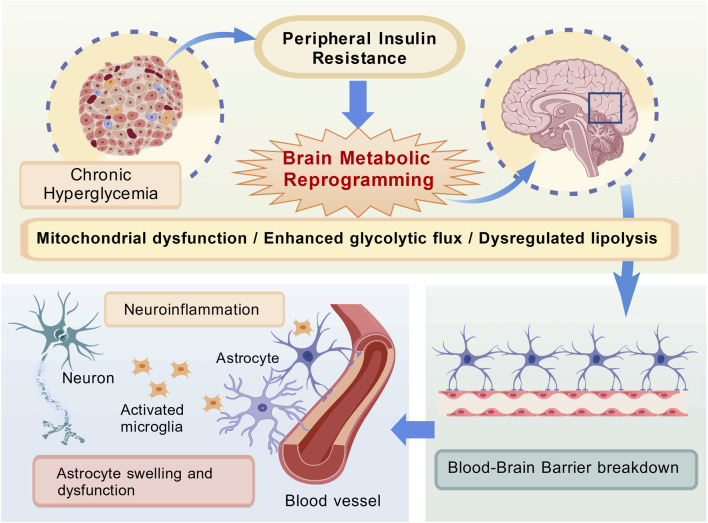
Brain Metabolic Reprogramming as a Central Hub Linking Peripheral Metabolic Stress to Neurodegenerative Pathologies. Peripheral metabolic stress (chronic hyperglycemia/insulin resistance) triggers brain metabolic reprogramming, which in turn drives neuroinflammation and BBB breakdown. These pathologies impair synaptic plasticity, promote tau hyperphosphorylation, and reduce cognitive function.

Oxidative stress activates several stress-responsive signaling pathways, including p38 MAPK, NF-κB, AGE/RAGE, JNK/SAPK, and protein kinase C, which directly modulate metabolic reprogramming (Evans et al., 2002). NF-κB signalling, for example, inhibits PGC-1α, the main orchestrator of mitochondrial biological development, thereby inhibiting oxidative phosphorylation and shunting metabolism to glycolysis (Abu et al., 2023). Similarly, the p38 MAPK signaling inhibited Akt-guided glucose absorption, thereby exacerbating the imbalance of neuronal energy (Entezari et al., 2022). In total, these disorders modify proteins, lipids, and DNA oxidatively, leading to caspeze-induced apprehensis and persistent neuritis. In particular, protein-oxidation worsens synaptic plasticity and memory alignment, contributing to the cognitive decline directly observed in DE (Dong et al., 2023) (Figure 1).

## Autophagy and mitophagy dysfunction

Autophagy is a cleanup path using lysosomes. It removes bad proteins and cell parts, key for cell balance (Dikic and Elazar, 2018; Galluzzi et al., 2017). Main control paths for autophagy include AMPK/mTOR and PI3K/Akt/mTOR chains. These cross with core metabolic reprogramming (Wang et al., 2023; Kim et al., 2002). Phage flux is usually measured by LC3 and p62. Persistent high p62° signals inhibit macrophage flow (Mizushima et al., 2010).

Mitophagy is a specialized form of autophagy that selectively target mitochondria for lysosomal degradation and play an important role in regulating inflammation, metabolic transformation, and cell reprogramming (Gustafsson and Dorn, 2019). Because neurons are specialized energy consumers, can accelerate their death even with light mitochondrial damage and force metabolic recombination, that macrophages are central tubes that connect metabolic disorders and neurodegenerative diseases A growing number of data indicate that this is the case (Fang et al., 2019).

Under physiological conditions, instantaneous energy stress triggers the AMPK/ULK1 signaling axis and activates a protective mitotic food with PINK1 stabilization and Parkin recruitment (Iorio et al., 2021). However, chronic metabolic disturbances, such as persistent high blood sugar, disrupt this time regulation. When AMPK calibration is impaired, the PINK one phosphorylation mode is no longer adaptive, resulting in Parkin mistranslation and progressive self-feeding failure (Guo et al., 2023). The resulting mitochondrial dysfunction led to a vicious cycle of self-continuation, in which defects in cell removal exacerbated the overproduction of ROS, further impair the PINK 1-mediated activation mechanism (Lazarou et al., 2015). This leads to irreparable mitochondrial rupture, which runs out of ATP reserves and accelerates the over-phosphorylation of tau proteins—a sign of synaptic degeneration (Toyama et al., 2016).

In a db/db mouse model, a simulation of T2DM due to obesity and hyperglycemia, hippocampal self-eating injury was demonstrated by a decrease in the LC3-II/I ratio and an increase in p62 accumulation (Guan et al., 2017; Li et al., 2017). T1DM has recorded a similar mitochondrial phagocytotic protective effect that reduces the accumulation of ataxia mitochondria in pancreatic beta cells, and a conservative mechanism between diabetic subtypes tends to be consistent with ataxia (Blagov et al., 2023).

Recent breakthroughs have shown that enhancing mitotic diet can suppress amyloid-beta plaque accumulation, reduce excessive phosphorylation of tau protein, and prevent synaptic loss during neurodegeneration. It has been demonstrated that pharmacological activation of mitotic food by supplementation with drugs such as urolithin A or NAD^+^ reverses the glycolysis dependence of neurons, restores oxidative phosphorylation, and improves cognitive function in diabetic models (Fang et al., 2017; Fang et al., 2014).

## Glycolytic flux and lipolysis dysregulation

Chronic hyperglycemia leads to pathological changes, characterized by “glycolysis overload and unscheduled glycolysis associated with hexalose kinases”. Unscheduled glycolysis means a state of metabolic disorder in which glycolysis fluxes are persistent and are affected by cellular energy demands. This process is characterized by too large a bundle of glucose passing through hexokinase-2 (HK2), but the downstream yeast degrading enzyme does not increase accordingly. This imbalance leads to an abnormal accumulation of yeast solution intermediates to bypass mitochondrial oxidation and promote the production of excessive lactate (Rabbani and Thornalley, 2024a). Lactic acid usually acts as the preferred oxidative fuel for neurons during acute metabolic stress, but its chronic overproduction exceeds the physiological threshold in hyperglycemia (Magistretti and Allaman, 2018). In neurons, this shift appears as a change from oxidative phosphorylation to aerobic glycolysis, similar to the Warburg effect. This change happens through increased HIF-1α activity, which raises levels of pyruvate kinase M2 (PKM2) and lactate dehydrogenase A (LDHA) (Traxler et al., 2022; Wei et al., 2023). Lactate changes from a useful energy source to a harmful substance: it increases the lactate-to-pyruvate ratio in the brain and separates glycolysis from mitochondrial oxidation (Tang, 2020). More glucose use leads to higher ATP consumption (Rabbani and Thornalley, 2024b; Wang et al., 2024). This broken lactate shuttle system prevents proper energy production, making neurons lose energy (Lee et al., 2025).

Lipid breakdown problems also speed up brain aging and cognitive loss. Research shows these problems cause free fatty acids (FFA) and lipoproteins to enter the brain, damaging the BBB. Studies found that clusters of Apolipoprotein E epsilon 4 allele (ApoEε4) turn on the CypA-MMP9 pathway (Banks and Rhea, 2021; Shen et al., 2023). This breaks down tight junction proteins and lets neurotoxic free fatty acids flood into the brain. The resulting lipid overload not only amplizes insulin resistance in neurons, but also builds a self-sustaining cycle of metabolic dysfunction (Zhao et al., 2017). The high blood sugar induced glycolysis flux directly intensified the malfunctions of lipolysis. Enhanced glycolysis generates glycerol-3-phosphate, which converts FFA esters to triglycerides and releases FFA permeated into the brain by elevated adipose triglyceride lipase (ATGL) (Pedretti et al., 2025; Byrns et al., 2024). In neurons and star-like colloidal cells, insulin resist inhibition of peroxisome proliferator-activated receptor α (PPARα) activity, inhibits fatty acid oxidation (FAO), and promotes lipid accumulation (Scheggi et al., 2022; Kumar et al., 2025).

**FIGURE 2:**
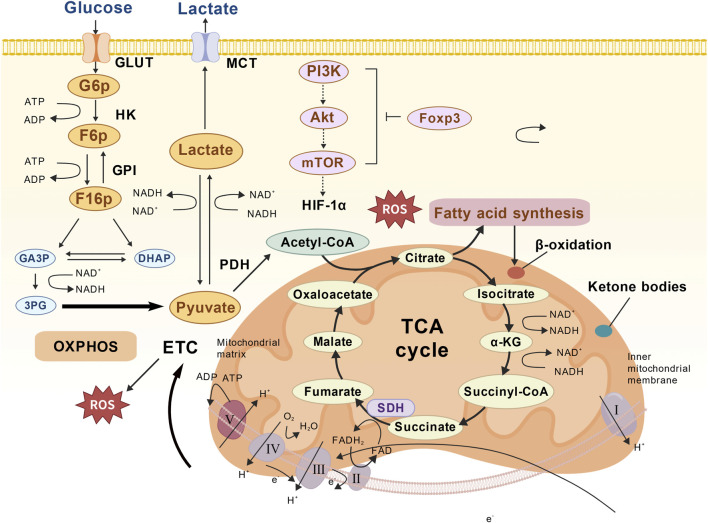
Putative Metabolic Reprogramming in DE: Interconnected Pathways of Glucose Utilization, Mitochondrial Oxidation, and Lipid Dysregulation. (1) Glucose Metabolism: Glucose uptake via GLUT (glucose transporter) and glycolysis, with pyruvate diverted to lactate (exported via MCT [monocarboxylate transporter]) under chronic hyperglycemia (Warburg effect); (2) Mitochondrial Dysfunction: Impaired oxidative phosphorylation (OXPHOS) due to ROS (reactive oxygen species) from the electron transport chain (ETC.), reducing ATP production; (3) Lipid Metabolism: Dysregulated fatty acid synthesis (from TCA cycle citrate) and β-oxidation, contributing to lipid accumulation.

The interaction between glycolysis and lipolysis produces a self-amplified pathological circulation in the DE. First, citric acid is transferred from the TCA cycle to the abinitio fat production mediated by acetyl-CoA carboxylase (ACC) to enhance the synthesis of palmitic acid. Palmitates then activate the mini-colloidal cell Toll-like receptor 4 (TLR4), triggering the release of pro-inflammatory cytokines (Kim et al., 2023). At the same time, lactate is released through the monocarboxylic acid transport protein, which activates neuron G protein-coupled receptor 81 (GPR81) and inhibits intracellular cAMP levels. This decrease in cAMP indirectly inhibits the activity of hormone-sensitive lipase (HSL) and captures lipids into the cell (Lee et al., 2025). Finally, the ROS oxidative low density lipoprotein (LDL) particles produced by overloading are glycosylated to form oxidized LDL (oxLDL). This in turn raised the sterol regulatory element-binding protein 1c (SREBP-1c), accelerating the synthesis of ceramides. The resulting increase in hippocampal ceramide levels directly results in synaptic damage and cognitive impairment (Goodman et al., 2024; Zhou et al., 2025) (Figure 2).

## Therapeutic strategies targeting metabolic reprogramming

Current evidence supports the targetability of therapy with pathological metabolic reprogramming to offset progression of DE (Cunnane et al., 2020; Guarente et al., 2024). GLP−1 receptor agonists (including liraglutide and semaglutide) improve neuronal insulin resistance by enhancing insulin-sensitive glucose utilization and inhibiting yeast fluke driven by hyperglycemia (Drucker et al., 2017). Clinical trials have shown that they reduce neuroinflammation and synaptic loss primarily by indirectly regulating peripheral intestinal insulin signals and steady glucose status (Wei et al., 2023; Li Z. et al., 2021). The exact involvement of HIF-1α/PKM2 signaling in neurons is not yet fully understood and further research is needed.

NAD^+^ boosters, exemplified by nicotinamide mononucleotide, address hallmark NAD^+^ depletion through the reactivation of sirtuins SIRT1 and SIRT3. This reactivation restores mitochondrial oxidative phosphorylation, enhances autophagic clearance of damaged organelles, and reduces oxidative damage in hippocampal neurons, thereby reversing cognitive deficits associated with accelerated brain aging (Fang et al., 2014; Butterfield and Halliwell, 2019; Zh et al., 2016). Emerging evidence demonstrates that supplementation with NAD^+^ or its biosynthetic precursors rescues high-glucose. It impaired keratinocyte proliferation and migration, expedites corneal re-epithelialization and sensory nerve regrowth in diabetic animals, and coincides with re-engagement of the SIRT1 pathway (Li Y. et al., 2021). Brain NAD^+^ regulation presents a tissue-specific pathway with different enzymatic controls, and these findings indicate that hyperglyceme-mediated NAD^+^ biosynthetic damage is a pathogen in diabetic tissue pathology.

AMPK activators exemplified by metformin operate through well-established mechanisms: inhibition of mitochondrial complex I activates AMPK, suppressing acetyl-CoA carboxylase-mediated lipogenesis and ceramide accumulation. It also activates PPARα to promote oxidation of fatty acid beta and relieve lipid-induced astrocytes stress (Bharath et al., 2020; Zhang et al., 2023). Clinical data have demonstrated the therapeutic effect of metformin in improving glucose control and reducing diabetic complications, but its direct impact on the specific lipolysis of human astrocytes requires testing (Dutta et al., 2023).

New evidence suggests that combinatorial targets of key metabolic nodes may enhance neural protection for age-related neurodegenerative diseases (Zhang et al., 2021). To restore brain metabolic stability and slow brain aging, a synergy of GLP-1 receptor agonists, NAD^+^ precursors and AMPK activators was proposed. The resulting metabolic recombination reverses the transformation of Warburg-like glycolytic shifts in senescent neurons, enhances macrophage lysosome function, and may reduce oxidative stress. It should be noted that the therapeutic potential of this combination must be balanced with clinical risk. Hypoglycemia due to combination of GLP-1RA and metformin and uncertain central nervous system interactions under tissue-specific NAD^+^ regulation (Xie et al., 2023; Gong et al., 2022). Targeted validation in diabetic encephalopathy models remains a necessary condition to assess the safety tradeoff of treatment efficacy (Na et al., 2025).

## Conclusion

This review identifies metabolic reprogramming as an important driver of DE cognitive decline and linked the major pathologic processes: brain insulin resistance, oxidative stress, defective autophagy, and dysregulated glycolysis/lipolysis, all of which contribute to accelerated brain aging. Novel treatments for metabolic reprogramming, such as GLP-1 receptor agonists, NAD^+^ boosters and AMPK activators, have shown hope in preclinical studies, but the specific mechanisms in terms of DE and clinical efficacy are unknown. Importantly, direct research into DE metabolic reprogramming is not yet sufficient and there are significant differences in our understanding of its causal effects and therapeutic potential. Future research should focus on the validation of these mechanisms and conduct clinical trials to develop effective methods for treating DE related dementia.

## Glossary

ACC: Acetyl-CoA carboxylase
AD: Alzheimer’s disease
AGE/RAGE: Advanced Glycation Endproducts/Receptor for AGEs
Akt: Protein kinase B (PKB)
AMPK: AMP-activated protein kinase
Apaf-1: Apoptotic protease-activating factor 1
ApoEε4: Apolipoprotein E epsilon 4 allele
ATGL: Adipose triglyceride lipase
ATP: Adenosine triphosphate
BBB: Blood-brain barrier
CaMKK2: Calcium/calmodulin-dependent protein kinase kinase 2
CREB: cAMP-response element-binding protein
CypA-MMP9: Cyclophilin A- Matrix metallopeptidase 9
DE: Diabetic encephalopathy
DM: Diabetes mellitus
FAO: Fatty acid oxidation
FFA: Free fatty acid
GLP-1: Glucagon-like peptide-1
GLP-1RA: GLP-1 receptor agonist
GLUT4: Glucose transporter type 4
GPR81: G protein-coupled receptor 81/Hydroxycarboxylic acid receptor 1
GRO: Growth-regulated oncogene (chemokine)
HK2: Hexokinase 2
HIF-1α: Hypoxia-inducible factor 1-alpha
HSL: Hormone-sensitive lipase
IFN-γ: Interferon gamma
IL-1β: Interleukin-1 beta
IL-6: Interleukin-6
iNOS: Inducible nitric oxide synthase
IR: Insulin receptor
IRS: Insulin receptor substrate
JNK/SAPK: c-Jun N-terminal kinase/Stress-activated protein kinase
LDHA: Lactate dehydrogenase A
LDL: Low-density lipoprotein
LPS: Lipopolysaccharide
MAP1LC3/LC3: Microtubule-associated protein 1A/1B-light chain 3
MAPK: Mitogen-activated protein kinase
MCP-1: Monocyte chemoattractant protein-1 (CCL2)
MMP: Matrix metallopeptidase
mTOR: Mechanistic target of rapamycin
**NAD** ^**+**^: Nicotinamide adenine dinucleotide (oxidized form)
NF-κB: Nuclear factor kappa-light-chain-enhancer of activated B cells
NO: Nitric oxide
Nrf2: Nuclear factor erythroid 2-related factor 2
oxLDL: Oxidized low-density lipoprotein
p62/SQSTM1: Sequestosome 1
PGC-1α: Peroxisome proliferator-activated receptor gamma coactivator 1-alpha
PI3K: Phosphatidylinositol 3-kinase
PKM2: Pyruvate kinase isoform M2
PPARα: Peroxisome proliferator-activated receptor alpha
RAGE: Receptor for Advanced Glycation Endproducts
ROS: Reactive oxygen species
SDHAF1: Succinate dehydrogenase complex assembly factor 1
SIRT: Sirtuin (Silent mating type information regulation 2 homolog)
SREBP-1c: Sterol regulatory element-binding protein 1c
T1DM: Type 1 diabetes mellitus
T2DM: Type 2 diabetes mellitus
TCA cycle: Tricarboxylic acid cycle (Krebs cycle)
TLR4: Toll-like receptor 4
TNF-α: Tumor necrosis factor-alpha
TXNIP: Thioredoxin-interacting protein
